# Filling Root Canals with an Improved Paraffin Compound*Abstract of a paper read before the St. Louis Dental Society, September 2nd, 1912, and printed in the Dental Review.

**Published:** 1913-01-15

**Authors:** Herman Prinz

**Affiliations:** St. Louis, Mo.


### FILLING ROOT CANALS WITH AN IMPROVED PARAFFIN COMPOUND.*

BY HERMAN PRINZ, A.M., M.D., D.D.S., ST. LOUIS, MO.

One of the most subtile operations in the routine procedure of conservative dentistry is the filling of root canals. In the great majority of cases, the successful completion of this operation requires an extraordinary amount of dexterity and an over-abundance of patience. Aside from the intricate technical requirements which are primarily based on a thorough knowledge of dental anatomy and which can only be mastered by diligent practice, the materials employed for such purposes are of equal importance. Regarding the physical and chemical nature of the various root filling materials, apparently the greatest diversity of opinion exists. Only recently a writer on this subject enumerated over two hundred and fifty odd compounds and methods of their application. Comparatively little experimental work has been done, clinical observations have been the guide posts largely for this operation. With the progress of our knowledge of dental pathology and its recent addition, bacteriology, the demands on the nature and functions of the substances employed as root canal obturators have materially increased, and, consequently, what at one time was considered a perfect root filling, as for instance, a plug made of leaf gold, is today considered wholly inadequate for the purpose.

### THE OBJECT OF FILLING ROOT CANALS.

The primary object of filling a root canal is to replace as perfectly as possible the artificially or pathologically destroyed pulp with a solid, unchangeable and inert sub-

*Abstract of a paper read before the St. Louis Dental Society, September 2nd, 1912, and printed in the Dental Review.

stance. If the canal is not filled, serum will seep into it from the apical tissues. The serum furnishes nutrient material for the micro-organisms present in the tubuli of a primarily infected root canal. In a sterile canal, endogenous infection may occur *via* the circulation as Faiszt has recently shown experimentally. This same mode of infection may take place in cases where the pulp of a sound tooth has died as a result of some traumatic insult. In both instances, secondary infection of the pericementum with its sequences will usually occur in due time. The specific materials used as root canal obturators should, according to Miller, Dunning and others, possess the following qualifications: 1) The material should be non-putrefactive; 2) It should possess permanent antiseptic qualities; 3) It should be easy of introduction; 4) It should not irritate the soft structures beyond the foramen; 5) It should not discolor the tooth substance; 6) It should not be porous and remain unchanged as to form; 7) It should, in case of necessity, be easy of removal; 8) As Mayrhofer has suggested, it should seal the dentinal tubuli and the apical foramina hermetically against bacterial invasion, and 9) if possible, it should offer some resistance to the roentgen rays so as to furnish an opaque shadow picture. To enumerate all the substances which have been advocated at one time or another as root filling materials and to criticize their respective use fulness is entirely outside of the scope of this paper. Merely to mention the most important groups of materials we may state that silk or cotton, either raw or purified, medicated or carbonized; asbestos, the cements, metals in the form of wire, foil, or as amalgams; gutta percha in its pure form or in its many modifications; wood points; semi-solid pastes, principally composed of zinc oxid combined with various antiseptics, and certain diverse substances, i. e., salol, paraffin waxes, resinous compounds, balsams, etc., have been used with more or less success. Clinical observations and laboratory experiments have demonstrated that out of all these suggested materials only a few comply with the demands.

Silk and cotton in their pure state, or in their many modifications, have always been failures as root filling materials. Among the various cements, the oxychlorid of zinc has been the most successful compound and such skilled operators as Jenkins, E. Kells, Jr., Ottolengui, and others, recommend it as being practically the most suitable material for this purpose. The introduction of cements into tortuous canals, is difficult, they are irritants if forced beyond the foramen and their complete removal from a root canal is often impossible. Metals, on account of their imperfect adaptation and natural resistance to manipulation in the minute canals have been practically discarded. Gutta percha, in the form of cones, in conjunction with its solution in chloroform or some of the essential oils and covered with zinc chlorid cement, as Webster and Cook have shown experimentally, are practically moisture proof, and, to some extent, prevent the ingress of bacteria. Cook is partial to a chloro-percha solution, claiming that a slow liberation of active chlorin takes place in a root canal filled with this compound, which acts as a powerful antiseptic. Other practitioners prefer a solution of gutta percha in eucalyptol or in cajuputol, myrtol or cineol.

Laboratory tests made with these compounds in glass tubes and in extracted teeth have demonstrated that, in some cases, a watertight filling can be made. At various times test fillings have been made with the gutta percha preparations before societies and it was invariably found that less than 50 percent of the fillings could be called "good" root fillings. Gutta percha in its many modifications or combined with the cements is principally employed in the United States and Canada as a root filling material, while in Europe semi-solid pastes are at present highly valued for such purposes. Experimental work with the latter compounds has proved that they are valueless, they prevent the ingress of moisture only temporarily. Wood points, artificially prepared, or in the form of natural thorns or as charcoal points, are porous and, consequently, are useless substances. Salol, when melted into a root canal, will be absorbed within a short time. Some of the balsams, i. e., Canada balsam, balsam of Peru, Balsamo del Deserto, concentrated solutions of sandarac and other resinous compounds are still lauded by many practitioners, especially balsam of Peru which has lately been recommended again. The solid resins possess many advantages over other compounds; their successful introduction into fine root canals, however, is very difficult. Of the various waxes, the insoluble hard paraffin has given most excellent results and it is about this last substance that the following discussion is concerned.

It is quite difficult to state who first advocated it for this purpose, most likely, however, we are indebted to Charles S. Tomes for its introduction. In 1883, he presented a paper before the Odontological Society of Great Britain entitled: Note upon a method of Filling Root Canals.

### CHEMISTRY OF PARAFFIN.

Paraffin, also known as hard paraffin, paraffin wax, is, according to the U. S. P., "a mixture of solid hydrocarbons, chiefly of the methane series, usually obtained by chilling and pressing the distallates from a petroleum having high boiling points, and purifying the solid pressed cake so obtained." The name paraffin was given to this substance by its discoverer, Reichenbach, who, in 1830, obtained it among the products of dry distillation of beach wood. At present it is usually obtained by purifying the waxy substance procured by distillation from petroleum or bituminous shales, or from natural deposits known as ozokerite (earth or mineral wax.)

Chemically, the paraffins correspond to the general formula $CnH2n+2$; the ultimate analysis yielding about 85 per cent carbon and 15 per cent hydrogen. Purified hard paraffin is a neutral, white inodorous and tasteless substance, having a micro-crystalline waxy appearance with a specific gravity ranging from about 0.8 to 0.9. It is insoluble in water and indifferent to the strongest acids, alkalies and oxydizing substances, hence its name, *parum affinis*—of little affinity. Chloroform, carbon bisulphid, gasolin, xylol, ether, oil of turpentine, olive oil and, to a very limited extent, warm alcohol will dissolve it. When melted it may be readily mixed in any proportion with fats, waxes, stearin, oils and resins. The melting point of paraffin is variable, it depends largely upon the origin of the material. The so-called soft paraffin, also known as petrolatum, vaselin, cosmolin, and by many other trade names, melts at about 104—108° F., while the melting point of the hard paraffins ranges from 113—176° F. Ceresin, the hardest of the paraffin series, has the highest melting point, about 176° F. Heated to about 456° F., it is volatilized without leaving a residue. Technically, paraffin is very largely used in the manufacture of candles and lubricating mixtures, as a preservative for meat, eggs, etc., and for water-proofing wood, paper and other materials. It is also used in large quantities as a base for chewing gum, for cheap candies, coating pills, etc.

### THE THERAPEUTICS OF THE PARAFFIN ROOT FILLING COMPOUND.

Paraffin as a root canal filling was introduced, as we have stated above, about 1883. In spite of its many advantages over other substances employed for such purposes it has not come into general use. The reasons for this we believe may be largely attributed to faulty technic, to the wrong kind of paraffin, and to the fact that the paraffin filling, in certain cases, will disappear from the root canal after a more or less prolonged period of time.

However, most observers, agree that root canals from which the paraffin had been absorbed were always found to be free from infection. The disappearing of the root filling is the point most vigorously objected to by those that have used it; if it can be overcome, paraffin seems to be the most suitable material for root filling purposes, or, expressed in the words of a professional friend: Prove to me that this danger does not exist and I shall be a most enthusiastic advocate of it. To explain this phenomenon we believe with Charles Tomes, that the ordinary paraffins, i. e. those having a melting point of less than 113° F., and the so-called base-plate paraffin combinations, which have been principally used for this purpose in former years, become absorbed by the tooth structure. If there should have been present a comparatively large foramen, some of the soft paraffin may have become displaced by ingrowing tissue. These deductions are in conformity with the results obtained by experimental work. If we place a tooth with an open empty root canal in melted soft paraffin, stained with an anilin dye, and keep it for some time at body temperature in an incubator, we find that on splitting the tooth, its tubules are filled with the colored paraffin. On the other hand, the hard paraffin, i. e., that which melts above 131° F., when subjected to the same treatment, is not absorbed into the tooth structure. If the high melting paraffin is previously dissolved in xylol and a tooth root is kept in this solution for a few days at body heat, its tubules also become filled with the solution. The changes which occur in the structure of paraffins of diverse melting points when injected into living soft tissues verify this fact. For these various reasons, it is imperative that the paraffin which is used for root filling purposes must have a melting point of not less than 131° F. Much higher melting paraffins offer no advantages; the high heat which is required for their liquefaction may cause injury to the soft tissues about the foramen and the necessarily high heated instruments are a constant source of danger to the patient and the operator. From our experimental work we have found that what is commercially known as "hard paraffin," melting point 133—136° F., gives the most satisfactory results. Since the correct melting point of the paraffin used for this work is of the utmost importance, it is invariably necessary to determine the melting point of every lot of paraffin used for this purpose.

Since paraffin is colorless, it is of advantage to incorporate some dye material but which must not stain the tooth structure. Incidentally, as Dunning has pointed out, this added substance should give "body" to the paraffin and it should make the paraffin plug fairly impermiable to the X-rays. Bismuth subnitrate, which is largely used for X-ray work in medicine and surgery, has been recommended as an admixture of Dunning to the extent of about 45 per cent. Such large quantities destroy the normal cohesion of the paraffin molecules; it becomes what is technically known as "short." Furthermore, bismuth subnitrate is too bulky and is readily decomposed by high heat and, therefore, it is not suited for combining with a high melting paraffin. We have experimented with a large number of metallic compounds and with pure metals in an extreme fine state of division. Merely to mention a few of the substances experimentally employed, we name the various bismuth salts, mercury sulphid (vermillion), hydrated iron oxid (crocus martis), zinc oxid, zirconium oxid, powdered lead, zinc, bismuth, electrically reduced metals, etc. We have finally found in the anhydrous bismuth oxid, $Bi_2O_3$, also known as bismuth trioxid, a substance which is well adapted for this purpose. Anhydrous bismuth oxid contains about 90 per cent of metallic bismuth, while the subnitrate contains only about 72 per cent, hence the greater resistance of the former substance to the roentgen rays. If 30 per cent of a dry, finely powdered bismuth oxid is added to the liquified hard paraffin, a smooth compound of a dull lemon yellow color is obtained which produces a distinct shadow picture when it is viewed with the fluoroscope.

The addition of antiseptics to insoluble substances, i. e., high melting paraffin, gutta percha, etc., which are used as root filling materials has practically very little value. Theoretically, at least, it seems improbable since antiseptic action means to become soluble, or to speak more correctly, the drug has to undergo ionization in the medium with which it is surrounded. We should remember that the pharmacologic action of a drug is manifested only when this drug enters the tissues as a liquid or in gas form. When a solid drug is introduced it necessarily follows, as we pointed out, that it must be soluble in the tissue fluids if its typical action is to be manifested. Since hard paraffin, gutta percha, and other similar substances are insoluble in the body juices, they prevent the solution of the drug or drugs which are dissolved in or mixed with them. If the insoluble root filling material contains a soluble antiseptic but offers no resistance to the invading surrounding fluids, i. e., cotton, wooden points, various cements, semi-solid pastes, etc., it will be exhausted in due time. This statement is sufficiently substantiated by clinical observation and experimental work. Inoculated plate cultures in which cones of sterile hard paraffin containing reasonable quantities of thymol, mercuric bichlorid, bismuth oxid, iodoform, etc., are suspended, show a more or less tiny zone of inhibition of bacterial growth within their immediate periphery. It is an indication that some of the antiseptic material deposited upon their surface has entered into a solution. But these small quantities of the antiseptic are too minute to be regarded as being of permanent value. No bacterial growth is observed upon the suspended pure sterile paraffin cones. The ordinary moulds, however, will grow on paraffin. If we wish to cover a root canal with a powerful antiseptic it seems more reasonable to apply this antiseptic separately or dissolved in a volatile medium prior to the insertion of the final root filling. Of the many powerful antiseptics which possess a relative permanent action, comparatively few answer our purpose. A normally liquid antiseptic is not as good suited as one which can be deposited in dry form and which slowly dissolved in the moisture present in the dentinal tubuli. Thymol dissolved in acetone answers the purpose well. It should be borne in mind that no fluid substance will enter the dentinal tubuli of a tooth root *in situ* at once on account of the present moisture; it requires a more or less prolonged period of time (days and weeks) to accomplish this osmotic interchange and it should also be remembered that experiments regarding the penetrability of antiseptics made with tooth roots out of the mouth do not represent normal conditions and are, therefore, misleading. From experimental work we are led to believe that acetone is a fluid which is especially suited as a carrying medium on account of its ready affinity for water, its solvent action upon fatty substances, its penetrating power, its hardening effect on tissues and its ready vaporization. Acetone, also known as pyro-acetic spirit, is a colorless mobile and volatile liquid having a characteristic ethereal odor and a pungent sweetish taste.

### THE PREPARATION OF THE PARAFFIN ROOT FILLING COMPOUND.

The formula of the paraffin root compounds reads as follows:

| | |
|---|---|
| Thymol | 2 parts. |
| Bismuth trioxid | 30 parts. |
| Hard paraffin melting point | 68 parts. |

Commercial paraffin contains many impurities, principally soluble chlorids and sulphates, water, and mechanical admixtures. To purify the paraffin, it is placed in a vessel containing water and boiled for about half an hour under constant stirring. After cooling, the cake of paraffin is removed, its lower part, which contains the insoluble impurities, is cut away and the process of boiling in fresh water is repeated. After the second cooling, the paraffin cake is thoroughly dried and placed in a porcelain capsule and heated to about 248° F. and kept at this temperature for about half an hour. This heat removes the present small quantities of water and incidentally sterilizes the paraffin. It is now removed from the fire, filtered through cotton cloth and slightly cooled off. Under constant stirring, the thymol is now added, and, after it has dissolved, the previously dried powdered bismuth trioxid. After the mixture becomes solid, it is slowly remelted on a water bath, well stirred, and cast into suitable molds.

### EXPERIMENTAL WORK.

When pure sterile paraffin is injected into the human body with ordinary precautions, it is tolerated by the tissues without further reaction, provided that the injected quantity is within reasonable limits (about 2 to 3 grams to the dose.) This statement is sufficiently substantiated by the rather extensive use of paraffin for prosthetic purposes in surgical practice. The same is true of the paraffin compound as suggested by us for the filling of root canals. When the semi-liquified paraffin compound is forced beyond the foramen, it molds itself about the end of the tooth and, on congealing, forms a solid cap over its apex. For this reason hard paraffin is the most satisfactory canal filling material in all cases where an open foramen, an incomplete calicfied root or a perforation of the root is present.

When soft paraffin is injected into the tissue, the changes which occur differ markedly from the above observations. The soft paraffin, i. e., those which melt below 106° F., are also incapsulated but the connective tissue fibres grow through the entire mass in all directions, and, in due time, as it has been stated by Erkstein, Delangre, Ligoric and others, this semi-organized paraffin becomes absorbed through the action of the leucocytes. The present extensive use of Beck's bismuth paste in surgery and dentistry, which contains approximately 70 per cent of soft paraffin, furnishes excellent proofs of this process of absorption. Not alone does the paraffin become absorbed but the tissue fluids dissolve the otherwise insoluble bismuth subnitrate and, as a consequence, severe bismuth poisoning has been observed in some cases. Hence, *we* emphasize again that a paraffin which is used as a root filling material must have a melting point above 131° F.

To test the impermeability of paraffin to water, innumerable tests were made. Thick-walled glass tubing, drawn out to capillary points, and extracted teeth were used in connection with various colored fluids, principally methylen blue solutions. When glass tubing is used for experimental work, its expansion and contraction under heat must be taken into consideration. The results of these various tests merely indicate that the paraffin or paraffin compound when melted into a root canal *lege artis* forms an absolute water-tight plug.

To ascertain the adaptability of paraffin compounds to the walls of root canals, the canals were prepared in the routine manner and filled with the paraffin compounds. The teeth were now placed in 50 per cent solution of hydrochloric acid and set aside in a warm place. The acid has to be renewed every day. Usually within three to five days the entire tooth substance is completely destroyed. The remaining root fillings are washed in water and kept in a dish filled with the same fluid. The root fillings float on the water and may be readily studied with a magnifying lens. Great care is required in handling these delicate paraffin castings, they will easily break. The root fillings present a perfect plastic picture of all the irregularities of the root canals. The paraffin compounds had flowed into all ramifications and irregularities of the canals, even the fine burr markings near the entrance of the canals could be observed. In studying these casts, it was noticed that the irregularity of the anatomic structure of the roots canals occurred with an astonishing regularity.

Regarding the relationship of pathogenic bacteria to hard paraffin we have stated above that the latter has no influence on the growth of these organisms and that it does not furnish a suitable nutrient medium for their further development. Paraffin is a hydrocarbon (not a fat) and, consequently, it is not subject to putrefaction.

In using the paraffin compound, or, in fact, any other material, for the filling of root canals, the question is often asked: What becomes of the air if forced beyond the foramen when filling a canal? This little air when forced beyond the foramen will be taken care of by the soft tissues, and since it is not forced into a vessel, no danger from air embolism is to be expected. That such small quantities of air, even if injected into a vessel, are absolutely free from danger, has been recently experimentally demonstrated by Blair and McGuigan.

### TECHNIQUE OF FILLING ROOT CANALS WITH THE PARAFFIN COMPOUND.

The sine qua non of a successful paraffin root canal filling is an absolutely dry root canal. To accomplish this end, certain physical procedures are in vogue, i. e. the hot air blast, the electrically heated root dryer, the heated wire, of which the Evans root dryer probably is the best known prototype, bibulous paper cones, cotton, etc. To facilitate the removal of moisture hygroscopic chemicals, i. e., alcohol, chloroform, ether and other substances, are often used in conjunction with the above enumerated means. These compounds, with the exception of alcohol, have little affinity for water and hence are of no practical value. As we have stated above, acetone, is well suited for this purpose. In drying out a root canal, it should be borne in mind that the removal of its natural moisture or any other fluid placed into it is well-nigh impossible with the much lauded hot air blast if its foramen is closed. A few trials on an extracted tooth or a glass tube drawn out to a fine solid point and filled with water or any other of the above enumerated fluids will readily convince one of its illusory conception. The fluid will move back and forth upon the elastic cushion of air confined in the end of the tooth or the tube, or, if no air is present the heated air blast will practically make no impression on the moisture column. The removal of the moisture is usually best accomplished by using bibulous paper cones in conjunction with the heated metallic root canal dryer. The secession of the hissing sound following the introduction of the hot wire indicates that the desired effect has been successfully achieved. In passing, it is well to remember that over-drying of the tooth structure is a dangerous procedure. If more or less of the water which holds the gelatinous matrix of the tooth in colloidal solution is removed by over-heating, that tooth is proportionately weakened against physical or chemical insults. Black, Cook and others have repeatedly called attention to this fact and it is well borne out by clinical observation.

After the root canal is freed from its moisture, it is flooded with acetone and dried again with the hot wire point. The object of this procedure is to remove every particle of moisture from obscure nooks. When a root canal which is kept continuously moist with a most annoying, persistent seepage is thoroughly dried out with acetone and the heated root dryer, the seepage will usually stop immediately. A wisp of cotton wrapped about a broach and dipped in pure paraffin oil (also known by many trade names, such as liquid alboline, cosmoline, oliphane, etc.) is now passed into the dry root canal and immediately followed by the hot compressed air blasts so as to uniformly coat the canal. Its object is to cover every accessible surface with a thin film of oil, in turn, facilitates the ready flow of the liquified paraffin compound into every available space. Too much oil must be carefully avoided. This lubricating of the canal constitutes an important factor in the correct technique of the paraffin root filling method. A cone of the prepared paraffin is now inserted into the canal and the heated root dryer passed along its side. By a gentle pumping motion the air is expelled and the semi-liquified paraffin is allowed to flow into the canal. The film of the previoulsy introduced paraffin oil and the semi-liquified paraffin compound possess great affinity for each other and whenever the oil film has been deposited the paraffin compound will readily flow. Care should be exercised not to overheat the paraffin compound. It is essential, however, to keep the root dryer fairly warm so as not to chill the sem-liquified paraffin when the dryer reaches the deeper portions of the canal. As the paraffin compound melts at less than 140° F., there is no danger of burning the patient with it. Sufficient paraffin compound is now added so as to completely fill the canal. It has been suggested to finally insert a gutta percha cone or a heated copper wire into the filled canal and leave it permanently in place. Such procedures insure a more perfect filling; they overcome the shrinkage of the paraffin in solidifying. Gutta percha cones as suggested by Dunning are especially well suited for this purpose; they act as a core and insure a more perfect adaptation of the softened paraffin to the irregularities of the root canal. In using these cones their extreme points are first cut off and they are then quickly pressed in the sem-liquid paraffin. Fine copper wires for such purposes are readily obtained by cutting suitable pieces from an electric light cord. On congealing of the paraffin compound a slight depression in the center of the filling near the entrance of the root canal will be noticed. This contraction is in conformity with the natural tendency of the hardening of the paraffin; it will always congeal from the periphery toward the center and thereby insures an unchangeable, permanent and absolute watertight sealing of the canal walls. The paraffin compound finally is covered with a layer of oxychlorid or oxyphosphate cement to form a solid foundation for the future permanent filling. If it should become necessary at some future time to remove the root canal filling, the introduction of the heated wire will readily liquify it; it can then be removed with bibulous paper cones, the broach, and if necessary, with a solvent, i. e. xylol.

The position of the patient in filling root canals of teeth in the lower jaw is self-explanatory; in filling the canals of the upper teeth, the chair is tilted backward so as to obtain a horizontal position of the upper teeth. As capillary attraction plays an important role in this procedure, the paraffin compound will readily follow the heated wire.

Regarding the instruments used as root pluggers for this work, any wire which will retain heat sufficiently long enough to melt the paraffin and which can be filed to a point fine enough to reach the smallest canals is suitable for this purpose. Silver or copper, or an alloy thereof, are good heat conductors and therefore the most suitable metals. A one-inch coil made of No. 10 or 12 copper wire, with a three-inch extension, filed to a fine point, answers the purpose fairly well, the Evans root canal dryer with its silver point is also very useful, though the best results are obtained with the electrically heated point. The heat, which can be perfectly gauged in the latter appliance, at once characterizes its superiority over the other instruments. The fine point of the root canal plugger must be able to carry a minimum temperature of 140° F.

In recapitulating the technique of root canal filling with the paraffin compound, the following points are the most important factors which must be rigidly observed:

Carefully drying out of the root canals.

Lubricating the canal walls with a thin film of paraffin oil.

Filling the canals with the prepared paraffin compound by means of a heated wire, using a pumping motion.

Covering of the finished root filling with a layer of cement.

During the last two years, the improved paraffin compound has been used extensively as a root filling material in our infirmary and by a number of practitoners. So far the results obtained have been exceedingly gratifying and we now take this opportunity of offering the material and the technique of its application to the profession at large for further trials.

### RESUME.

Hard paraffin, having a melting point of 133—136° F. with the addition of 30 per cent of bismuth trioxid and 2

per cent of thymol possesses the following qualities as a root canal filling material:

First. It is not putrefactive.

Second. It is sterile and slightly antiseptic.

Third. It is easily introduced.

Fourth. It is absolutely non-irritating to the soft tissues; when forced beyond the foramen of a temporary or permanent tooth or through a perforated root, it is borne by the soft tissues without the slightest reaction.

Fifth. It does not discolor the tooth structure; it possesses a distinct yellow tint which makes it readily discernible to the eye.

Sixth. It is not porous and is unchangeable; it produces an absolute permanent water-tight filling.

Seventh. It is easily removed.

Eighth. It will seal hermetically the dentinal tubuli and the foramina against bacterial invasion.

Ninth. It is opaque to the Roentgen rays.

*Dental Review*, Dec. 1912.

